# Development of stem cell‐derived microfluidic models of the blood‐brain barrier in Alzheimer’s disease

**DOI:** 10.1002/alz.089413

**Published:** 2025-01-03

**Authors:** Lily E Takeuchi, Craig A Simmons

**Affiliations:** ^1^ University of Toronto, Toronto, ON Canada

## Abstract

**Background:**

Drug discovery efforts in neurological diseases, such as Alzheimer’s disease (AD), have had particularly poor outcomes due to the lack of models that capture the cerebral vasculature. There is an unmet need to develop models that capture the physiological challenge of overcoming the blood‐brain barrier (BBB) and impacts of blood flow‐induced shear stress. In this work, we use a microfluidic platform to model the cerebral vasculature in familial AD (fAD) using patient‐derived brain endothelial‐like cells (BECs) and neurons.

**Method:**

Stem cells derived from a patient with fAD (PSEN‐2 N141I) and an unaffected control line were differentiated into BECs and neurons. BECs were cultured statically or exposed to 12 dynes/cm^2^ of shear stress for 72 hours prior to assessment of barrier permeability using a fluorescent tracer, monocyte adhesion, and efflux transport function using receptor‐inhibition assays.

**Result:**

BECs derived from the patient with AD (AD‐BECs) demonstrate reduced capacity for efflux transport by p‐glycoprotein (p‐gp), breast cancer resistant protein (BCRP), and multidrug resistant protein (MRP‐1) compared to controls (fControl‐BECs, **p = 0.0015, ***p = 0.0004, ***p = 0.0002, respectively). Under shear stress conditions, these impairments were not present, suggesting shear stress may play a protective role in maintaining efflux transport function. AD‐BECs show enhanced susceptibility to cytotoxic effects of Aβ42 oligomers between concentrations of 1.25 ∼ 20 µM with cell viability reduced by 19 ∼ 24%. AD‐BECs exhibit increased monocyte adhesion (1.9‐fold; **p<0.01) which was reduced by the application of shear stress in both lines (AD: ***p<0.001; fControl: **p<0.01).

**Conclusion:**

This in‐depth characterization of patient‐derived BECs in both static and physiologically relevant shear conditions demonstrates the cerebral vasculature in fAD may be impaired in areas of drug transport, immune cell trafficking, and cytotoxicity, particular in the absence of shear stress as occurs in cerebral hypoperfusion.

